# Excellent response to chemotherapy post immunotherapy

**DOI:** 10.18632/oncotarget.20030

**Published:** 2017-08-08

**Authors:** Ashish D. Dwary, Samip Master, Abhishek Patel, Constance Cole, Richard Mansour, Glenn Mills, Nebu Koshy, Prakash Peddi, Gary Burton, Dalia Hammoud, Kavitha Beedupalli

**Affiliations:** ^1^ Department of Medicine, Division of Hematology-Oncology, Louisiana State University Health Sciences Center, Shreveport, LA, USA

**Keywords:** excellent response, chemotherapy, immunotherapy

## Abstract

**Introduction:**

Immunotherapy in the form of immune checkpoint inhibitors has changed the landscape of cancer treatment. Newer monoclonal antibodies are coming up and are being tested in various cancers during different stages of treatment. With the increasing use of immune checkpoint inhibitors in the management of various types of cancers, the question is raised as to what next can be offered to a patient who has progressed on this newer treatment. Does Sequence matter? There have been reports of improved responses to chemotherapy after immunotherapy in the form of vaccines. Here we present a case series of 6 patients who progressed on immunotherapy with immune checkpoint inhibitors after initial modality of treatment (chemotherapy/radiation), subsequently received chemotherapy with excellent response.

**Methods:**

We have a cohort of six patients who had disease progression on second line Immunotherapy for solid or hematological malignancies and had ECOG < 2. All these patients received third line salvage chemotherapy. Three patients had metastatic head and neck cancer, 2 had non-small cell lung cancer (NSCLC), and one had T -cell rich B- cell lymphoma. Prior review and approval were obtained from our institutional review board.

**Results:**

All patients had an excellent response to chemotherapy in third line setting, after immune checkpoint inhibitors and most of them achieved a complete response.

**Conclusion:**

Targeting cancer with chemotherapy after failure of immunotherapy is a valid option and can lead to better response rates and PFS which may lead to OS. This effect may be secondary to immunotherapy removing the inhibition exerted by tumor cells or other immune cells initially followed by cytotoxic chemotherapy mediated killing of tumor cells.

## INTRODUCTION

Immunotherapy class of drugs are redefining how we treat cancer. Tumor cells evade immune response by blocking the T-cell mediated apoptosis [1]. Immunotherapy with PD-1 and PDL-1 ligand inhibitors work via removing the inhibition exerted by tumor cells or other immune cells. US Food and Drug Administration has approved immunotherapy mostly in the second line setting after progression on conventional chemotherapy for multiple stage IV cancers (Head and neck cancer [2,3], Non-small cell lung cancer [4]^,^ [5], Bladder cancer [6-10], Renal cell cancer [11] and melanoma [12,13]). In NSCLC (Non-small cell lung cancer), FDA has approved PD-1 inhibitor in first line setting in patients with high PDL-1 expression (PDL-1 expression >50 %) [14]. PD-1 inhibitors have been approved in selected cases of Hodgkin’s Lymphoma after progression on multiple lines of chemotherapy [15]. A sizeable number of patients progress on frontline chemotherapy and immunotherapy and have limited options going forward. Response to Immunotherapy is related to PDL-1 expression in various cancers. Hyperprogression of disease has been reported after use of immunotherapy in head and neck cancer [16]. Initial studies have shown increased clearance of bound anti PD-1 antibodies on T cells by tumor-associated macrophages resulting in ineffective response and progression on Immunotherapy [17]. Chemotherapy use with Immunotherapy has resulted in better response rate in the first line setting in Non-small cell lung cancer [18]. Here we present a case series of six patients who progressed on immune checkpoint inhibitor therapy and subsequently received chemotherapy with excellent response.

## CASE 1

**Figure 1:**
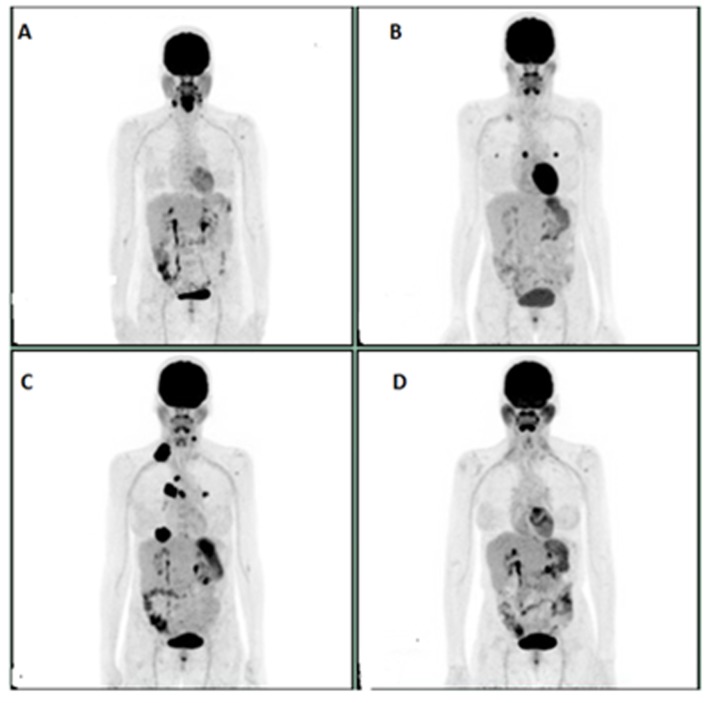
**A:** Initial PET scan prior to chemoradiation**. B**: PET scan prior to initiation of Pembrolizumab. **C**: PET scan showing progression after 4 cycles of Pembrolizumab. **D**: Complete response after 6 cycles of weekly PCC.

A 61-year-old female patient who was initially diagnosed with stage IV A squamous cell cancer of supraglottis, P-16 negative in November 2015. She was treated with concurrent chemoradiation with weekly Cisplatin and had a complete response in the head and neck region based on positron emission tomography (PET) scan done in June 2016. However, it showed new bilateral pulmonary nodules and right subcarinal LN and EBUS biopsy confirmed Metastatic squamous cell carcinoma. Patient was started on Pembrolizumab (anti PD-1 receptor or PD-1 inhibitor) in September 2016 which was continued until December 2016. PET scan done after four cycles of Pembrolizumab showed disease progression. Options including palliative care were discussed. She opted for more treatment options. She was started on weekly PCC (Paclitaxel/Carboplatin and Cetuximab) at that point of time given ECOG <2. She tolerated the chemotherapy very well without any Grade 3 or Grade 4 toxicities. Subsequent scans after six cycles of weekly PCC showed complete response (Figure 1A-1D).

## CASE 2

**Figure 2:**
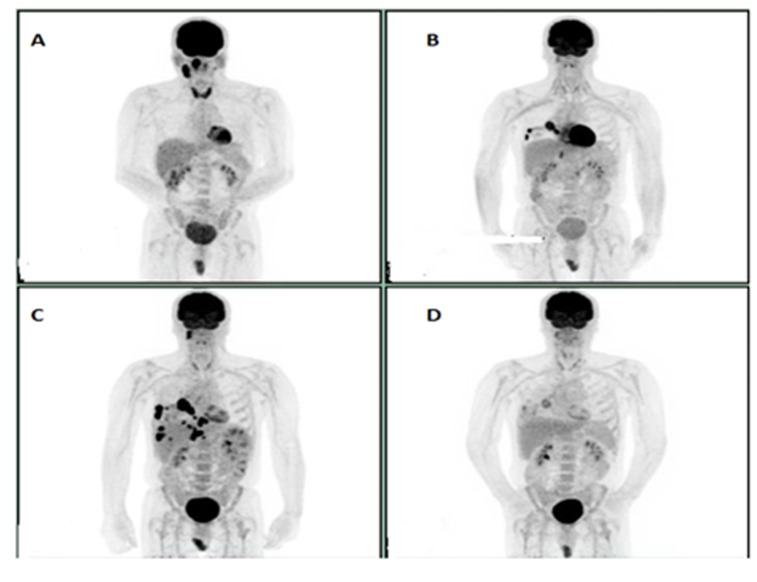
**A:** Initial study**. B**: PET scan prior to initiation of Pembrolizumab. **C**: PET scan after 4 cycles of Pembrolizumab showing progression. **D**: Virtual Complete response after 6 cycles of PC post Pembrolizumab progression.

A 54-year-old white male with Stage IV A Squamous cell cancer of right tonsil P-16 positive (T2N2bM0) in August 2012. The patient was initially treated with concurrent chemo-radiation with Cisplatin 100 mg/M2 every three weeks. The patient completed chemo-radiation and was lost to follow up until 7/2014. PET scan done at that time showed no local disease. However, in the interim, he had developed an intense right hilar focus and biopsy of the hilar LN 12R on August 2014 confirmed Squamous cell carcinoma, basaloid type. On immune- histochemistry it was positive for P16 and P63 and negative for CD56 and synaptophysin. Given excellent performance status, he was started on Carboplatin (AUC 5 on day 1) and 5FU (1000 mg/m2 on day 1-4) with weekly Cetuximab (400 mg/m2 loading followed by 250 mg/m2) for two cycles. The PET scan post treatment on March 2015 showed sustained response in head and neck region, but persistent and slightly more prominent small subpleural nodularity in lateral right midlung concerning for new metastasis. He was started on Methotrexate which was continued until September 2016 and was stopped due to disease progression in the lungs. Immunotherapy with Pembrolizumab was started on on October 2016 and remained on it for a year. Patient progressed on Pembrolizumab after which he was switched to chemotherapy with weekly Paclitaxel and Cetuximab. The PET scan done after six cycles chemotherapy showed virtual complete metabolic response with minimally FDG avid lesions in right mid and lower lung field (Figure 2A-2D).

## CASE 3

**Figure 3:**
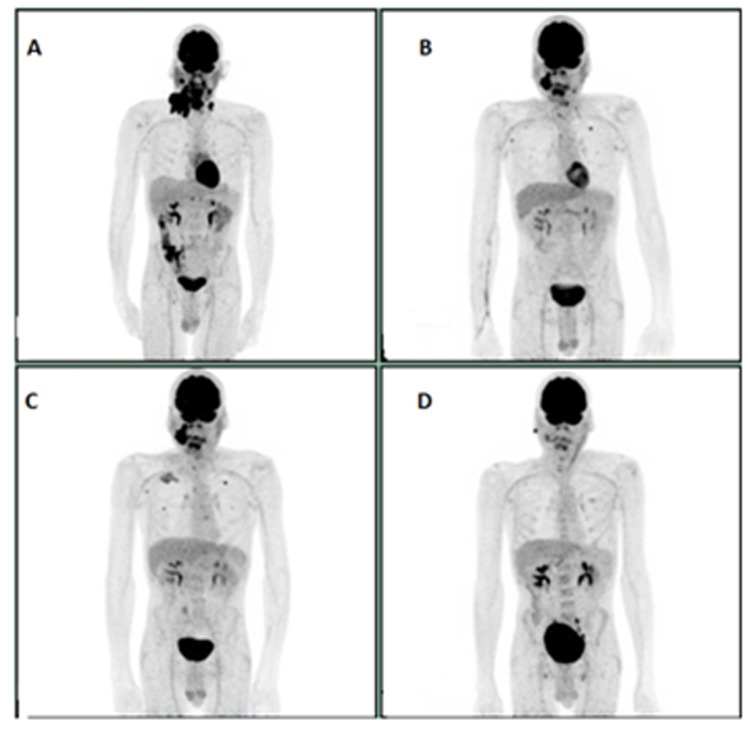
**A:** Initial study**. B:** PET scan after 4 cycles of Pembrolizumab. **C**: PET scan after 6 cycles of Pembrolizumab showing progression. **D**: Virtual Complete response after 6 cycles of PCC post Pembrolizumab progression.

A 54-year old male who was initially diagnosed with stage IV A squamous cell cancer of hypopharynx in November 2013 was started on induction chemotherapy with Docetaxel/Cisplatin and 5-Fluorouracil (DCF) given bulky disease and subsequently received concurrent chemoradiation with weekly Carboplatin (AUC 1.5) and Paclitaxel (50 mg/m2) with complete response. He later underwent right neck dissection for disease recurrence in the neck in April 2016. PET scan in August 2016 showed recurrent disease in the right neck with left upper lobe FDG avid lung nodule. Biopsy confirmed Squamous cell carcinoma. He was started on Pembrolizumab and received a total of 6 cycles. PET scan after six cycles showed disease progression. Palliative care versus weekly PCC were discussed with the patient. Patient opted for weekly PCC. PET scan performed after six cycles of weekly PCC showed great response and patient is currently receiving maintenance Cetuximab (Figure 3A-3D).

## CASE 4

**Figure 4:**
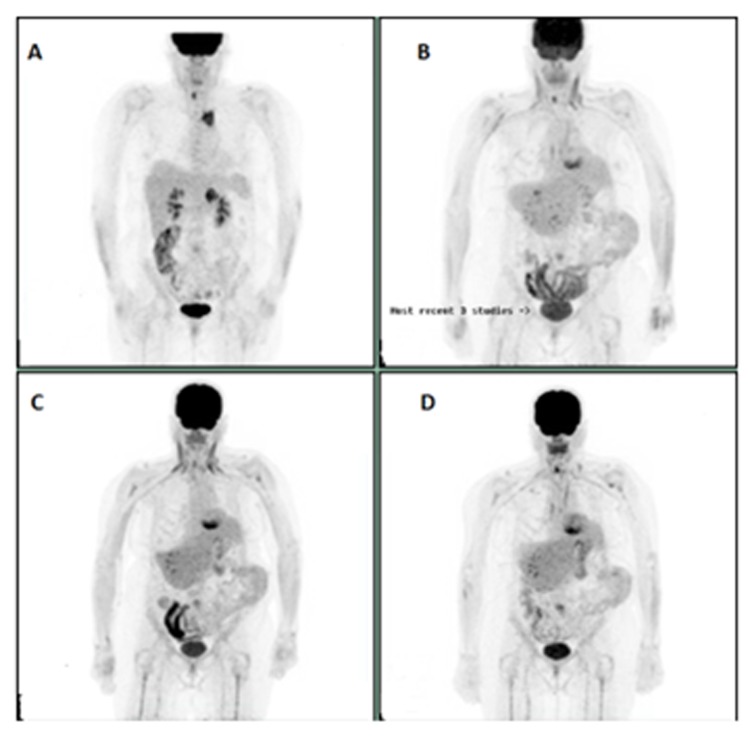
**A:** Initial study**. B**: Mild progression of the right adrenal lesion on Nivolumab. **C**: PET scan showing progression of right adrenal lesion further. Docetaxel started after this EPT scan. **D**: Resolution of the right adrenal lesion on PET scan.

A 60-year-old woman who was initially diagnosed with oligometastatic non-small cell lung cancer (NSCLC) (EGFR and ALK-negative) in 2010. Biopsy confirmed mucinous adenocarcinoma. Initial PET scan showed metastatic lung adenocarcinoma. The patient was started on Carboplatin, Pemetrexed, and Bevacizumab for four cycles and later maintained on Pemetrexed and Bevacizumab. Given issues with tolerance, stable findings on imaging, patient was given chemo holiday from July2011 to December 2011. Computerized tomography (CT) scan done in December 2012 showed disease progression and single agent Pemetrexed was restarted. Restaging scans in March 2012 showed disease progression and was started on Docetaxel. Patient received Docetaxel till August 2012 and was later held due to stable disease on imaging. Reimaging in April 2014 showed complete resolution of the adrenal lesion. Patient received definitive radiation therapy to the lung lesion. Unfortunately, patient had recurrence with isolated adrenal lesion on PET scan done in October 2014 for which she underwent adrenalectomy and the pathology from this was consistent with metastatic mucinous adenocarcinoma with negative margins. Patient developed new cerebellar lesion in July 2015 which was resected. Imaging done in January 2016 demonstrated a new right adrenal lesion, and subsequently the patient was started on Nivolumab. The patient had an excellent response to Nivolumab but eventually progressed in November 2016 for which he was started on Docetaxel and received it from December 2016 to February 2017. PET /CT scan done in February 2017 showed resolution of all FDG avid lesions (Figure 4A-4D). Patient currently is under observation and remains in remission.

## CASE 5

**Figure 5:**
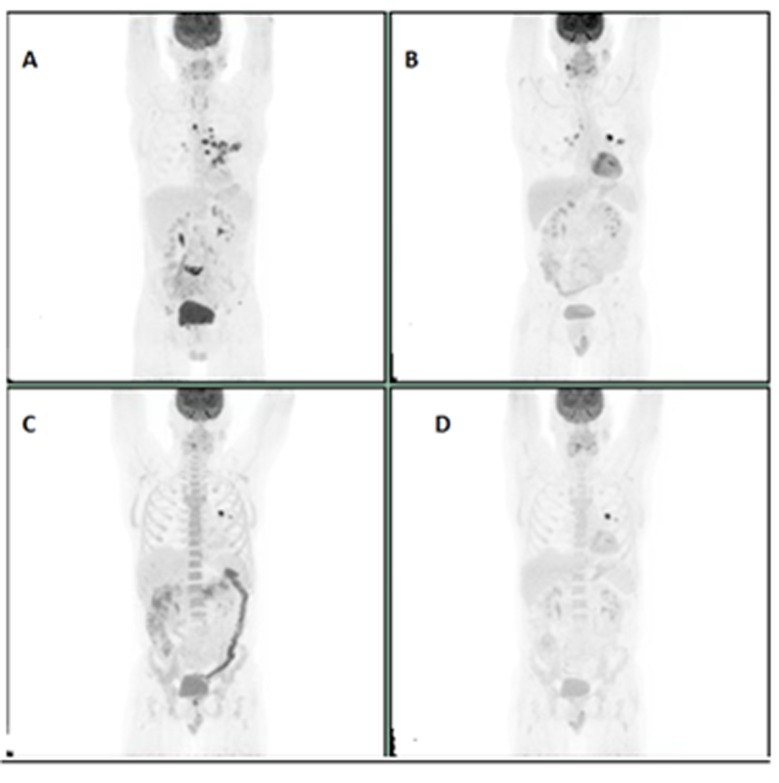
**A:** Initial PET scan at time of diagnosis**. B**: Patient with slight disease progression on Nivolumab. **C**: new lesion while patient on Nivolumab and as such it was stopped and patient was started on Docetaxel. **D**: significant response to Docetaxel.

A 50-year-old male was diagnosed with stage IV lung adenocarcinoma with spinal metastasis in September 2015. EGFR/ALK and ROS 1 were negative. Patient had extensive disease based on PET scan. He was also found to have brain metastasis. Patient underwent surgery for cord compression, this was followed by XRT to the vertebrae and whole brain radiation. He was started on Carboplatin and Pemetrexed from November 2015 to January 2016. He had some response to treatment, however couldn’t tolerate chemotherapy given issues with cytopenias, nausea and vomiting requiring multiple hospital admissions. He was subsequently switched to Nivolumab in February 2016. He eventually progressed on Nivolumab in November 2016. Patient was started on Docetaxel in December 2016 and PET scan done in February 2017 showed significant improvement with almost complete resolution of Right hilar, right mediastinal and right upper neck lesions with only some residual activity in Left lung suprahilar and perihilar regions ( Figure 5A-5D).

## CASE 6

**Figure 6:**
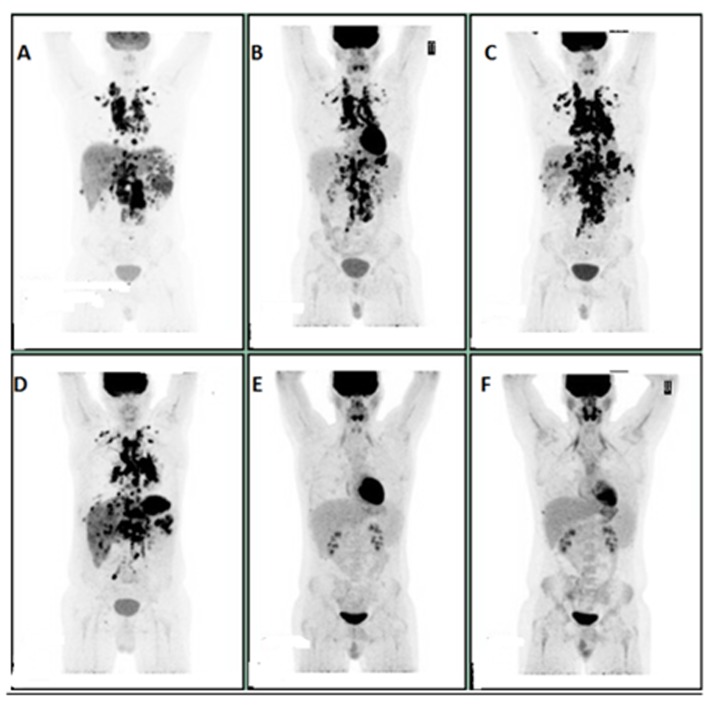
**A:** Initial scan in February 2016. **B**: In April 2016, after three cycles of R-CHOP. **C**: In June 2016, after five cycles of R-CHOP. **D**: In September 2016, after five cycles of Nivolumab. **E**: In December 2016, after three cycles of GDP. **F**: In May 2017, after five cycles of GDP

A 21-year-old male presented with persistent cough, weight loss, night sweats. PET scan in February 2016, showed intensely FDG avid lymphadenopathy in the lower neck, supraclavicular, chest and abdominal regions strongly suspicious for lymphoma. Left supraclavicular lymph node biopsy confirmed T cell rich B cell lymphoma and he was started on R-CHOP (Rituximab, Cyclophosphamide, Daunorubicin, Vincristine, and Prednisone). PET scan after five cycles of R-CHOP showed progressive disease on both sides of the diaphragm. The treatment was subsequently changed to Nivolumab on July 2016 and repeat PET scan in September 2016 after five cycles of Nivolumab revealed mixed response. The scan showed a considerable amount of FDG avid lymphadenopathy on both sides of the diaphragm. Some of the previously noted osseous lesions were improved, but there was more prominent involvement of liver, spleen and pleural and pericardial effusions were noted. Given concern for Nivolumab induced pneumonitis, Nivolumab was held, and the patient was started on prednisone in September 2016. He had a pericardial window and pathology from biopsy was positive for T cell rich B cell lymphoma. Patient was then started on salvage GDP (Gemcitabine, Prednisone, and Cisplatin) by the end of September 2016. A Pet scan after three cycles of GDP in December 2016 showed excellent metabolic response with virtual complete resolution of previously noted nodal and extranodal lesions and pericardial effusion, however with persistent bilateral pleural effusions (Figure 6A-6F). He completed five cycles of GDP and is being evaluated for autologous transplant.

**Table 1:** Summary of treatment for all cases.

	Primary Diagnosis	First Line Treatment/setting	Second/Third and subsequent line of chemotherapy prior to Immunotherapy.	Immunotherapy and PDL-1 expression.	Chemotherapy following Immunotherapy progression.
Case 1	Head and neck Cancer	Definitive Concurrent chemoradiation. Weekly Cisplatin 40 mg/m^2^ with radiation (total radiation dose of 70 Gray)	N/A	Pembrolizumab 200 mg every 3 weeks until progression. PDL-1 expression unknown.	Weekly PCC. (Paclitaxel/Carboplatin and Cetuximab). Carboplatin AUC 2 Paclitaxel 80 mg/m^2^ and Cetuximab 400 mg/m^2^ followed by 250 mg/m^2^ weekly.
Case 2	Head and neck cancer	Definitive Concurrent chemoradiation. Cisplatin 100 mg/m^2^ every 3 weeks for 3 doses with radiation (total radiation dose of 70 Gray)	**Second line:** Carboplatin (AUC 5 on day 1) and 5FU (1000 mg/m^2^ on day 1-4) with weekly Cetuximab (400 mg/m^2^ loading followed by 250 mg/m^2^) for two cycles. **Third line:** Methotrexate 40 mg/m^2^ weekly	Pembrolizumab 200 mg every 3 weeks until progression. PDL-1 expression unknown.	Weekly PCC. (Paclitaxel/Carboplatin and Cetuximab). Carboplatin AUC 2 Paclitaxel 80 mg/m^2^ and Cetuximab 400 mg/m^2^ followed by 250 mg/m^2^ weekly.
Case 3	Head and neck cancer	**Induction regimen:** **Day 1:** Docetaxel 75mg/m^2^ IV + cisplatin 75mg/m^2^ IV, **plus** **Days 1–5**: 5-FU 750mg/m^2^/day continuous IV infusion for 5 days.	Definitive concurrent chemoradiation with weekly Carboplatin (AUC 1.5) and Paclitaxel (50 mg/m^2^). Total dose of radiation 66 Grey.	Pembrolizumab 200 mg every 3 weeks until progression. PDL-1 expression unknown	Weekly PCC. (Paclitaxel/Carboplatin and Cetuximab). Carboplatin AUC 2 Paclitaxel 80 mg/m^2^ and Cetuximab 400 mg/m^2^ followed by 250 mg/m^2^ weekly.
Case 4	Non-small cell lung cancer	Carboplatin (AU 6) Pemetrexed (500 mg/m^2^), and Bevacizumab (15mg/m2) every 3 weeks for four cycles	**Maintenance therapy:** Pemetrexed (500 mg/m^2^), and Bevacizumab (15mg/m2) every 3 weeks. **Third Line:** Docetaxel 75mg/m^2^ every 3 weeks.	Nivolumumab 240 mg every 2 weeks until progression. PDL-1 expression unknown.	Docetaxel 75mg/m^2^ every 3 weeks.
Case 5	Non-small cell lung cancer	Surgery for cord compression and palliative radiation therapy to spinal cord and WBRT. Carboplatin (AU 6) Pemetrexed (500 mg/m^2^), every 3 weeks.	N/A	Nivolumumab 240 mg every 2 weeks until progression. PDL-1 expression unknown.	Docetaxel 75mg/m^2^ every 3 weeks
Case 6	T- cell rich B-cell Lymphoma	**First line:** R-CHOP: Rituximab 375mg/m^2^, Cyclophosphamide 750mg/m^2^ IV + doxorubicin 50mg/m^2^ IV bolus + vincristine 1.4mg/m^2^ IV bolus (max dose 2mg) Prednisone 100mg orally 5 days every 3 weeks.	N/A	Nivolumumab 240 mg every 2 weeks until progression. PDL-1 expression unknown.	GDP (Gemcitabine, Prednisone, and Cisplatin) **Days 1 and 8:** Gemcitabine 1000mg/m^2^ IV over 30 minutes **Days 1–4:** Dexamethasone 40mg orally **Day 1:** Cisplatin 75mg/m^2^ IV .

## DISCUSSION

The emergence of immunotherapy has changed the landscape of management of various solid and hematologic malignancies offering a potential for a durable response and survival. Malignant cells evade immune response by different mechanisms. Tumor cells secrete various chemokines such as CXCL12 which recruits regulatory T (Treg) cell and myeloid-derived suppressor cells in tumor microenvironment [19]. The release of IL-10, transforming factor beta and vascular endothelial factor from these cells results in inhibition of cytotoxic T cells and dendritic cells with resultant Immune evasion [20,21]. Upregulation of PD-1 on T cells or PDL-1 expression on tumor cells can cause evasion of the immune-mediated cytotoxic killing of malignant cells and tumor cells can downregulate MHC (major histocompatibility complex) antigen and hence T cell recognition [22]. Various stimulatory and inhibitory pathways play an intricate role in T cell-mediated killing of tumor cells.

Previous studies have shown the immunosuppressive effects of traditional cytotoxic chemotherapy. Cytotoxic chemotherapy can lead to cellular destruction and enhance the immune response against the tumor [23,24]. Chemotherapy is still used in majority of solid and hematologic malignancies. Various chemotherapies has been shown to exert immune-reactive effects such as upregulation of MHC class molecules or tumor antigens causing increase tumor antigen presentation [25,26]. Chemotherapy has also been shown to decrease the number of immunosuppressive cells in the tumor microenvironment such as regulatory T cells or myeloid-derived suppressor cells (MDSC), thereby increasing helper T-cell accumulation at the tumor site [27,28]. A Japanese study done on animal models showed that chemotherapy delivered after immunotherapy in the form of viral immunogene therapy augments anti-tumor efficacy. It resulted in increased number of antigen-specific CD 8+ T-cells systemically and within the tumors [29].

Recently few studies have shown an increase in the level of PDL-1 expression on tumor cells after treatment with chemotherapy [30,31]. The combination of chemotherapy with Immunotherapy in NSCLC has shown improved response rate in first line setting and has been recently approved by FDA [18]. Various phase 1 studies with other immunotherapy and chemotherapy combination have shown similar results [32]. There has been a case report showing response with the use of Nab-Paclitaxel and Pembrolizumab combination, in a patient with metastatic malignant melanoma who progressed after combination immunotherapy with Nivolumab and Ipilimumab [33]. Malignant melanoma has been considered to chemoresistant cancer, but there has been a report of excellent response to Dacarbazine and Cisplatin combination chemotherapy showing a good response in a patient with malignant melanoma who was previously treated with immunotherapy [34].

A Retrospective analysis of stage IV lung cancers presented at ELCC in May 2017 by Sache Roths Child et al. showed that patients who get salvage chemotherapy are 30 % more likely to achieve partial response if they have been pretreated with PD1 /PDL-1 inhibitors compared to the ones who were not [35].

Tumor microenvironment (TME) plays a critical role in tumor cells survival. Stratification of tumor microenvironment based on the presence of PDL-1 and tumor infiltrating lymphocytes (TIL) has been proposed [36]. Type I microenvironment shows the presence of both TIL and PDL-1 and as such presence of adaptive immune resistance. Immunotherapy will benefit the most in this subclass. Type II TME is characterized by the absence of PD-L1 and lack of TILs. The presence of PD-L1 and absence of TIL described Type III TME. Without TIL it is unlikely that blocking PD-L1 will lead to a T-cell response. Type IV TME tumors contain TILs but lack PD-L1, indicating the role of other suppressor pathways in promoting immune tolerance [37].

Given great response rate in various solid and hematologic malignancies in our case series in third line setting, we hypothesize that initial chemotherapy may have increased the PDL-1 / Neo antigen expression on cancer cells which are crucial for immune response. PD-1 inhibitors use subsequently may have blocked the PD-1 and PDL-1 tumor evasion. Treating these patient with conventional chemotherapy post immunotherapy, might make tumors more sensitive to the effects of chemotherapy. This may be attributed to its effects on decreasing number of immunosuppressive cells in the tumor microenvironment such as regulatory T cells or myeloid-derived suppressor cells (MDSC), thereby increasing helper T-cell accumulation at the tumor site which exert anti tumor effect by generating and maintaining immune responses.

Based on this case series, chemotherapy can yield excellent response in third line setting in patients who progress on immunotherapy. Our study has very few patients, and larger studies have to be done to confirm this data. The combination of Immunotherapy and chemotherapy and its sequencing should be studied in the context of tumor microenvironment.
